# Serum Interleukin‐35 Levels in Oral Squamous Cell Carcinoma and Salivary Gland Tumors: Implications for Early Detection and Prognosis

**DOI:** 10.1155/bmri/6091357

**Published:** 2026-02-01

**Authors:** Maryam Zahed, Sara Maroofi, Jannan Ghapanchi, Bijan khademi, Abbas Ghaderi, Mohamad Javad Fattahi, Niloofar Hayati, Fardad Khoubani

**Affiliations:** ^1^ Oral and Dental Disease Research Center, Department of Oral and Maxillofacial Medicine, School of Dentistry, Shiraz University of Medical Sciences, Shiraz, Iran, sums.ac.ir; ^2^ Department of Oral and Maxillofacial Medicine, School of Dentistry, Shiraz University of Medical Sciences, Shiraz, Iran, sums.ac.ir; ^3^ Department of Ear, Nose, Throat, School of Medicine, Shiraz University of Medical Sciences, Shiraz, Iran, sums.ac.ir; ^4^ Shiraz Institute for Cancer Research, School of Medicine, Shiraz University of Medical Sciences, Shiraz, Iran, sums.ac.ir; ^5^ Student Research Committee, School of Dentistry, Shiraz University of Medical Sciences, Shiraz, Iran, sums.ac.ir

**Keywords:** interleukin-35, oral squamous cell carcinoma, salivary gland tumors

## Abstract

**Background:**

Oral cancer, particularly oral squamous cell carcinoma (OSCC), is a major global health concern, with late‐stage diagnoses significantly lowering survival rates. Salivary gland tumors (SGTs), though less common, pose diagnostic challenges due to their varied presentation. This study investigates the role of interleukin‐35 (IL‐35) in OSCC and SGTs, aimed at assessing its potential as a biomarker for early detection and prognosis.

**Methods:**

A cross‐sectional study was conducted, including 65 OSCC patients, 65 SGTs patients, and 50 healthy individuals as a control group. Inclusion criteria included age over 18 years, negative HPV confirmation, and histopathologically confirmed cancer diagnosis (SGT or OSCC). Blood samples were collected from all participants. Serum IL‐35 levels were measured using the ELISA kit.

**Results:**

IL‐35 levels varied significantly between the groups (*p* = 0.002), with the lowest levels detected in the SGT group. Pairwise comparisons revealed that IL‐35 levels were significantly lower in the SGTs patients (5.45 ± 6.03 pg/mL) compared to both the OSCC group (9.21 ± 10.81 pg/mL, *p* = 0.002) and the control group (10.50 ± 11.77 pg/mL, *p* = 0.004). IL‐35 levels were significantly lower in patients with malignant SGTs (5.45 ± 6.03 pg/mL) compared to healthy controls (*p* = 0.002). There were no significant differences in IL‐35 levels between benign and malignant SGTs (*p* = 0.133). In OSCC patients, IL‐35 levels did not significantly differ from those in the control group (*p* > 0.05). Furthermore, OSCC tumor characteristics, including tumor origin and lymphatic involvement, showed no significant correlation with IL‐35 levels (*p* > 0.05).

**Conclusions:**

The study indicates that IL‐35 levels are notably reduced in SGTs compared to healthy individuals. However, no significant difference was observed between benign and malignant SGTs. The absence of significant correlation in OSCC patients suggests that IL‐35 may have a minor role in this type of cancer, highlighting the potential need for other biomarkers to improve early detection.

## 1. Introduction

Oral cancer ranks as the 13th most prevalent cause of death globally, with approximately 170,000 fatalities reported in 2020. Among the various forms of oral cancer, oral squamous cell carcinoma (OSCC) stands out as the most frequently encountered [1–3]. The development of sensitive biomarkers is essential for the early detection of OSCC, facilitating timely intervention and allowing for more personalized and effective treatment strategies [4].

Salivary gland tumors (SGTs) are a heterogeneous group of neoplasms categorized into benign and malignant forms, each varying in prevalence and demographic distribution across different populations [5–9]. Fine‐needle aspiration cytology remains the primary diagnostic method due to its high sensitivity and specificity for detecting masses [10–13]. Early detection of these tumors before they progress to advanced disease or malignant transformation can have a significant impact on treatment outcomes and survival [14].

Interleukin‐35 (IL‐35), a recently discovered cytokine within the interleukin‐12 family, is predominantly produced by regulatory T cells (Tregs) and is crucial for immune system modulation, particularly in the context of cancer [15]. Elevated levels of IL‐35 have been linked to adverse outcomes (e.g., disease progression, lymph node metastasis, and higher grades) in various malignancies, such as prostate cancer, pancreatic ductal adenocarcinoma, and non‐small‐cell lung cancer (NSCLC) [16, 17]. While the complete mechanisms underlying IL‐35’s actions remain to be fully clarified, it is known to activate the STAT3 signaling pathway. This activation is believed to inhibit T cell proliferation and facilitate immune evasion by tumors [18].

Previous research has established connections between various interleukins, such as IL‐6 and IL‐8, and OSCC [19, 20]. Additionally, IL‐33, CD73, and gene polymorphisms have been explored as potential markers for distinguishing between different types of SGTs [21–23]. The unique role of IL‐35 in inducing immune tolerance (unlike inflammatory cytokines such as IL‐6 or IL‐8) makes it an attractive target in OSCC and SGTs. Given its established association with poor prognosis in other epithelial cancers (e.g., pancreatic and lung) and the prevalence of Treg‐dependent immunosuppression in oral tumors, investigation of IL‐35 could reveal novel mechanisms of immune evasion and provide a more specific biomarker for early diagnosis and prognosis of these tumors. Both SGT and OSCC occur in the oral and maxillofacial region and require similar clinical workflows for diagnosis (e.g., imaging and biopsy). Furthermore, no study has yet investigated the diagnostic potential of IL‐35 in head and neck tumors.

However, there is a notable gap in the direct investigation of IL‐35 in the context of oral and salivary gland cancers. This study aims to address this gap by evaluating IL‐35 levels in patients with these cancers and comparing them to healthy controls. The findings are expected to enhance the understanding of IL‐35’s role in tumor development, immune modulation, and disease progression, potentially identifying new biomarkers for diagnosis and management.

## 2. Material and Methods

### 2.1. Patients

This cross‐sectional study included three groups of patients referred to Khalili hospital, Madar‐o‐Koodak Hospital, and Shiraz Dental School, Shiraz, Iran from April 2020 to April 2021.

The first and second groups were a SGT group and an OSCC group. The initial diagnosis for both groups was based on clinical symptoms, with a final confirmed diagnosis established for all cases based on histopathological examination of biopsy specimens. The third group consisted of healthy individuals referred to Shiraz Dental School for routine dental check‐up who did not require any surgical or non‐surgical treatment at the time of sampling. Inclusion criteria of the cases included age over 18 years, histopathologically confirmed cancer diagnosis (SGT or OSCC), and negative HPV confirmation. Exclusion criteria for the patients included the presence of concurrent tumors, immunological or genetic disorders, insufficient blood samples, a history of infectious or inflammatory diseases within the past 3 months, and any prior treatment for the tumor, including surgery, chemotherapy, or radiation therapy before the start of the study. The control group was selected from healthy individuals matched for age and gender with the patient groups. Individuals with a family history of cancer, autoimmune, or genetic diseases in first‐degree relatives were excluded from the control group. Furthermore, individuals with a history of infectious or inflammatory diseases within the past 3 months were also excluded from the control group.

This study was conducted after obtaining approval from the Ethics Committee of Shiraz University of Medical Sciences (IR.SUMS.DENTAL.REC.1400.089 and IR.SUMS.DENTAL.REC.1400.088). All patients provided written informed consent after being fully informed about the study procedures.

### 2.2. Sample Collection

From all participants, 5 cc of venous blood was collected in EDTA anticoagulant tubes. The samples were then coded and transferred to the laboratory for serum separation and stored at −80°C. For serum separation, blood was transferred into sterile screw‐capped tubes (within 30 min post‐collection) and allowed to clot at room temperature (25°C) for 30–45 min. The clot was gently separated using a sterile spatula, followed by centrifugation at 600 rpm for 20 min at 4°C to prevent hemolysis. The supernatant serum was aliquoted into 2‐mL cryovials using disposable Pasteur pipettes, avoiding clot disturbance. All samples were stored at −80°C within 2 h of collection to ensure cytokine stability. Freeze‐thaw cycles were strictly avoided. IL‐35 serum levels were measured using a Human IL‐35 ELISA Kit (Invitrogen, Thermo Fisher Scientific, United States; Cat. No. EEL067), following the manufacturer’s instructions. This ELISA Kit was selected for its high specificity in detecting the bioactive IL‐35 heterodimer (EBI3/p35), validated in peer‐reviewed studies with a sensitivity of 9.375 pg/mL and minimal cross‐reactivity with analogous cytokines (e.g., IL‐12 and IL‐27). All procedures were conducted by examiners blinded to the identity of the participants and their samples.

### 2.3. Statistical Analysis

The results are presented as mean ± standard deviation (SD) for quantitative variables and as frequency and percentage for categorical variables. The Kolmogorov–Smirnov test was used to assess the normality of the data distribution. Categorical variables were compared using the Chi‐square test. Quantitative variables were compared using either one‐way ANOVA or the Kruskal–Wallis test, depending on data distribution. Pairwise comparisons were conducted using the Mann–Whitney *U* test. Data analysis was performed using SPSS statistical software Version 27 (SPSS Inc., Chicago, IL), with a significance level set at 0.05.

## 3. Results

**Figure 1:**
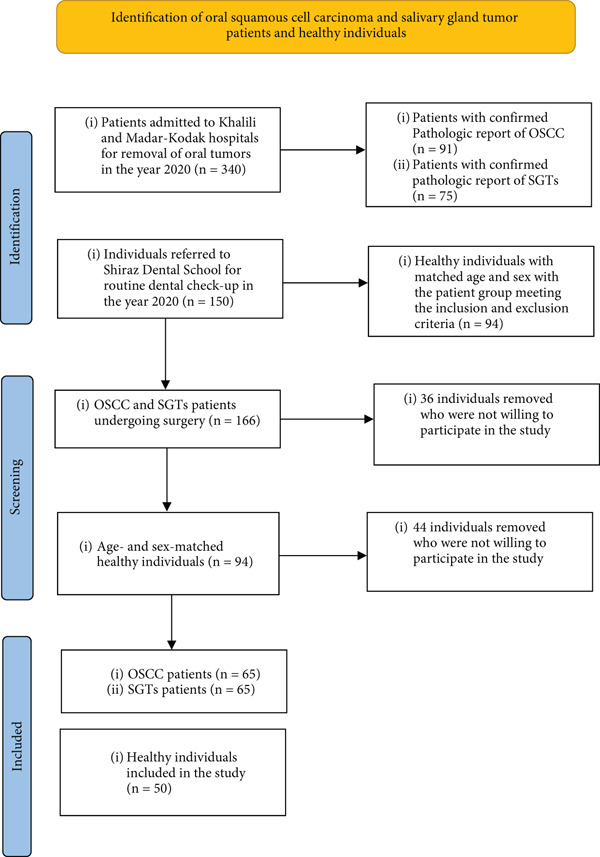
Flow chart of the patients and control subjects included in the study. OSCC, oral squamous cell carcinoma; SGTs, salivary gland tumors.

The study sample collection is explained in Figure 1.

The demographic characteristics of patients did not differ significantly between the study and control groups with respect to age (one‐way ANOVA, *p* = 0.067) and gender (Chi‐square test, *p* = 0.968). Among SGT patients, the most common tumor location was the parotid gland (58.5%), and the predominant pathological subtype was pleomorphic adenoma (50.8%), followed by adenoid cystic carcinoma (16.9%) and mucoepidermoid carcinoma (13.8%). In the OSCC group, the vast majority of tumors (89.1%) were located in the tongue (Table 1).

**Table 1:** Demographic and clinical characteristics of the studied groups.

		**Control**	**OSCC**	**SGTs**	**p** **value** ^∗^
Age (year)		54.06 ± 13.13	58.80 ± 15.54	53.26 ± 14.17	0.067
Gender	Male (*N*)	25 (50.0%)	32 (49.2%)	31 (47.7%)	0.968
	Female (*N*)	25 (50.0%)	33 (50.8%)	34 (52.3%)	
SGT tumor location	Parotid	—	—	38 (58.5%)	—
	Submandibular	—	—	12 (18.5%)	
	Sublingual	—	—	2 (3.1%)	
	Minor salivary glands	—	—	13 (20.0%)	
SGT pathological subtypes	Pleomorphic adenoma	—	—	33 (50.8%)	—
	Mucoepidermoid carcinoma	—	—	9 (13.8%)	
	Adenoid cystic carcinoma	—	—	11 (16.9%)	
	Acinic cell carcinoma	—	—	3 (4.6%)	
	Carcinoma ex pleomorphic adenoma	—	—	2 (3.1%)	
	Adenocarcinoma (not otherwise specified)	—	—	1 (1.5%)	
	Other malignant tumors	—	—	4 (6.2%)	
	Other benign lesions	—	—	2 (3.1%)	
OSCC tumor location	Tongue	—	57 (89.1%)	—	—
	Other oral cavity sites	—	7 (10.9%)	—	

Abbreviations: OSCC, oral squamous cell carcinoma; SGTs, salivary gland tumors.

^∗^Significant level of < 0.05.

**Figure 2:**
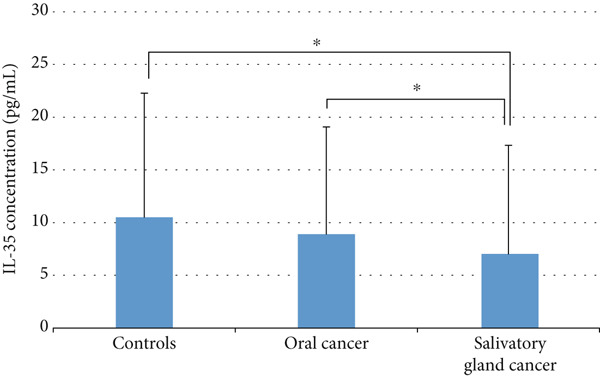
Evaluation of IL‐35 level (pg/mL) in patients and healthy subjects. Oral cancer: oral squamous cell carcinoma. Significant level of < 0.05.

In contrast, the Kruskal–Wallis test identified a significant difference in IL‐35 levels among the groups (*p* = 0.002). Pairwise comparisons conducted using the Mann–Whitney test revealed that IL‐35 levels in the salivary cancer group were significantly lower than those in both the control group (*p* = 0.004) and the OSCC group (*p* = 0.002) (Figure 2).

By dividing individuals in all three groups into two age categories with a cut‐off point of 50 years, the Mann–Whitney test results indicated no significant relationship between IL‐35 levels and age in the control group (*p* = 0.141), OSCC group (*p* = 0.540), or SGT group (*p* = 0.256). Additionally, the comparison of IL‐35 levels between genders revealed no significant association in the OSCC group (*p* = 0.376) and the SGT group (*p* = 0.890). However, in the control group, IL‐35 levels were significantly higher in men compared to women (*p* = 0.035) (Table 2).

**Table 2:** IL‐35 level in relation to gender and age in the studied groups.

		**Control group (pg/mL)**	**OSCC (pg/mL)**	**Salivary gland cancer (pg/mL)**
Age	50 years	15.43 ± 16.81	13.07 ± 16.44	9.17 ± 15.59
	50 years	8.39 ± 8.25	7.18 ± 5.34	5.91 ± 6.09
*p*		0.141	0.540	0.256
Gender	Male	13.26 ± 14.19	7.97 ± 7.47	7.50 ± 13.32
	Female	7.75 ± 8.11	9.81 ± 12.29	6.58 ± 6.70
*p*		0.035 ^∗^	0.376	0.890

Abbreviation: OSCC, oral squamous cell carcinoma.

^∗^Significant level of < 0.05.

SGT patients included 33 cases (50.8%) of benign tumors and 32 cases (49.2%) of malignant tumors. A Kruskal–Wallis test analysis of IL‐35 levels across controls, benign, and malignant SGTs revealed a significant difference (*p* = 0.006) (Table 3). Although the difference was significant between the three groups, subsequent pairwise comparisons using the Mann–Whitney test indicated no significant difference in IL‐35 levels between patients with malignant and benign SGTs (*p* = 0.133). Furthermore, IL‐35 levels did not significantly differ between healthy individuals and those with benign tumors (*p* = 0.094). In contrast, patients with malignant SGTs exhibited significantly lower IL‐35 levels compared to healthy controls (*p* = 0.002). In terms of tumor origin, 52 cases (80%) involved major salivary glands, while 13 cases (20%) involved minor salivary glands. The Kruskal–Wallis test revealed a statistically significant difference in IL‐35 levels when comparing the control group with patients harboring major and minor SGTs (*p* = 0.011) (Table 3). Pairwise analysis using the Mann–Whitney test demonstrated no significant difference in IL‐35 levels between patients with major and minor SGTs (*p* = 0.460). However, IL‐35 levels were significantly lower in patients with major SGTs (*p* = 0.017) and minor SGTs (*p* = 0.010) compared to the control group.

**Table 3:** Relationship between IL‐35 levels and characteristics of salivary gland tumors and OSCC.

			**N**	**Mean (pg/mL)**	**Standard deviation**	**p** **value** ^∗^
Control			50	10.50	11.77	0. 006
Salivary gland cancer	Tumor status	Benign	33	8.52	13.15	
		Malignant	32	5.45	6.03	
	Glands of origin	Minor	13	4.46	2.14	0.011
		Major	52	7.66	11.42	

OSCC	Tumor location	Tongue	57	9.21	10.81	0.938
		Other oral cavity sites	7	6.75	1.96	
	Lymphatic involvement	No	28	9.21	9.70	0.941
		Yes	24	9.79	13.08	

Abbreviation: OSCC, oral squamous cell carcinoma.

^∗^Kruskal–Wallis test: significant level of < 0.05.

Regarding OSCC, the analysis of IL‐35 levels based on tumor location revealed no significant difference (*p* = 0.938) between the tongue and other oral cavity sites (buccal mucosa, floor of mouth, and palate) (Table 3). Similarly, IL‐35 levels did not significantly differ between patients with or without lymphatic involvement (*p* = 0.941) (Table 3).

**Figure 3:**
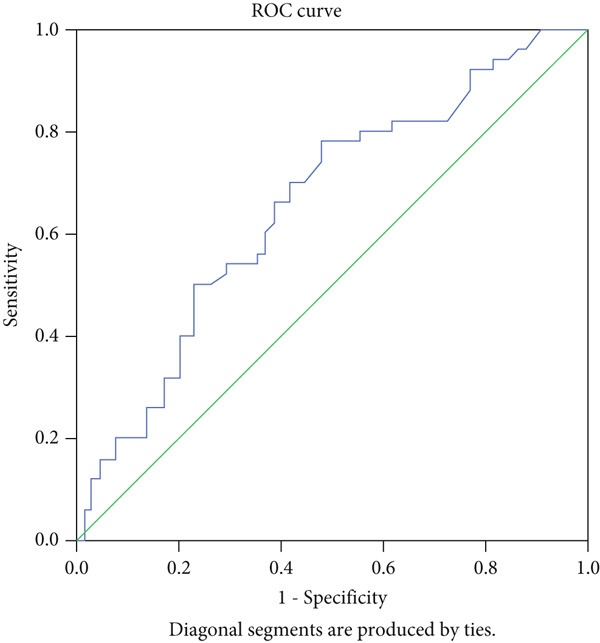
ROC curve for the diagnostic value of IL‐35 in predicting malignant SGTs.

To evaluate the diagnostic value of IL‐35 in distinguishing malignant SGTs from nontumorous conditions, a receiver operating characteristic (ROC) curve analysis was performed. The area under the ROC curve (AUC) was 0.65, with a 95% confidence interval ranging from 0.556 to 0.757, indicating a weak predictive ability of IL‐35 levels for differentiating malignant tumors from the control group. The IL‐35 cut‐off value identified in this study was 5.34, which was statistically significant (*p* = 0.004). At this threshold of 5.34, the test demonstrated a sensitivity of 70% and a specificity of 41%, as shown in Figure 3.

## 4. Discussion

In this study, we investigated serum IL‐35 levels in patients with SGTs and OSCC. IL‐35 levels in patients with SGTs were significantly lower than those in healthy individuals and patients with OSCC. Neither age nor gender had a significant impact on IL‐35 levels in cancer patients. Additionally, IL‐35 levels did not differ between patients with benign and malignant SGTs. However, the significantly reduced IL‐35 levels in patients with malignant tumors, compared to the control group, alongside the lack of significant difference between benign tumor patients and healthy controls, suggest that low IL‐35 levels may be indicative of malignant SGTs. The findings indicate that an IL‐35 level below 5.34 can differentiate malignant SGTs from nontumorous individuals, with a sensitivity of 70% and specificity of 41%. This diagnostic value is modest, indicating that IL‐35 may not be a strong predictor of SGT malignancy. The tumor origin (major or minor salivary glands) was not associated with IL‐35 levels. In OSCC patients, IL‐35 levels were not significantly different from those in the control group. Furthermore, OSCC tumor characteristics, including tumor origin and lymphatic involvement, showed no significant correlation with IL‐35 levels. Prior to this study, IL‐35 in head and neck cancers had been examined in only three studies, focusing on laryngeal squamous cell carcinoma [24] and nasopharyngeal tumors [25, 26]. Of these, only one study measured serum IL‐35 levels, and it was a case‐series report without a healthy control group for comparison. Therefore, our study is the first to measure and compare serum IL‐35 levels in a cohort of patients with SGTs and OSCC against a healthy control group. Given the critical need for biomarkers in these cancers, particularly for rare entities like SGTs, our findings provide a valuable foundation for future research into the immune microenvironment of head and neck tumors and the diagnostic utility of IL‐35.

IL‐35 is a unique anti‐inflammatory cytokine unlike other proinflammatory cytokines of the IL‐12 family [27]. Its activity across various tumor types has been shown to correlate with tumor progression, including factors like tumor size, lymph node involvement, and metastasis in cancers such as pancreatic ductal adenocarcinoma, breast cancer, and NSCLC. Elevated IL‐35 expression in tumor tissues and increased plasma levels in several malignancies are associated with poor prognosis, particularly in relation to tumor metastasis [28].

Other disorders are also associated with reduced IL‐35 levels in the literature [29, 30]. Reduced levels of IL‐35 are believed to play a role in the abnormal immune responses commonly seen in rheumatoid arthritis (RA). This suggests that IL‐35 may have a protective function in preventing autoimmune diseases and related disorders [29]. Another autoimmune disease linked to decreased IL‐35 levels is Sjögren’s syndrome (SS), an inflammatory condition primarily affecting the salivary glands. In primary Sjögren’s syndrome (pSS), the salivary glands’ function is significantly impaired. This is attributed to disruptions in innate immune pathways within the glandular epithelium, involving mechanisms like the nuclear factor‐*κ*B pathway, the inflammasome, and interferon signaling [30]. Patients with active pSS often exhibit lower serum IL‐35 levels. By analyzing sorted cells from blood and salivary gland biopsies of pSS patients and healthy controls, B lymphocytes were identified as a potential source of IL‐35. This finding supports the theory that regulatory B (Breg) lymphocytes might be crucial, though not the sole contributors, to IL‐35 production. However, in patients with active pSS, these cells are less effective [31]. As observed in the present study, low levels of IL‐35 observed in SGTs can be attributed to the same mechanism seen in the salivary glands of SS patients with lowered functions. This notion is further supported by a recent study in a dental context, which reported significantly lower salivary IL‐35 levels in patients with gingivitis and periodontitis compared to healthy individuals [32].

Recent studies have shown that IL‐35 can counteract lipopolysaccharide (LPS)‐induced endothelial dysfunction by suppressing endothelial‐to‐mesenchymal transition (EndMT). Since EndMT contributes to endothelial dysfunction, which is pivotal in conditions like sepsis, IL‐35 might play a protective role by preventing such dysfunctions, potentially aiding in the management of sepsis and related disorders [33]. The mechanism underlying the progression of SGTs in relation to low levels of IL‐35 remains unknown, but further research is warranted in this matter.

Research predominantly indicates that IL‐35, as a cytokine, promotes tumor growth and metastasis. However, there is also evidence suggesting that IL‐35 may act as an anticancer cytokine or a factor limiting tumor metastasis. Several studies, which differ from our findings, have reported elevated IL‐35 levels in various tumors. For instance, a study by Tao et al. demonstrated that IL‐35 exacerbates leukemia by enhancing the proliferation of Treg cells and inhibiting CD4 + CD25+ Teff cells [34]. Similarly, a study by Wang et al. found that in advanced gastric cancer, there is a significant increase in IL‐35‐producing B cells, which may contribute to disease progression [35]. A previous study by Aggarwal et al. reported increased transcriptional expression of IL‐35 in peripheral Treg cells from OSCC patients [36]. Similarly, a study by Wang et al. demonstrated that a high density of tumor‐infiltrating Tregs, which can be a source of IL‐35, was a strong independent predictive factor for the development of synchronous secondary cancers in patients with SCC, further supporting the immunosuppressive and protumoral role of the Treg/IL‐35 axis in this region [37]. Another study in 2018 showed that breast cancer cells (BCCs) express and secrete IL‐35, with elevated levels of IL‐35 in BCCs being closely associated with poor patient prognosis and serving as an independent adverse prognostic factor for breast cancer [38]. Furthermore, a 2020 study reported that in hepatocellular carcinoma, high levels of IL‐35 are present in both serum and tumor tissue, influencing the balance between Treg and cytotoxic T cells and playing a critical role in immune suppression [39]. The discrepancy between these studies and the present study may primarily be attributed to the different types of tumors investigated. Our study specifically examines SGTs and OSCC. Furthermore, the majority of the cited studies focused on histochemical and tissue analyses, without considering serum levels of IL‐35.

Conversely, a study reported decreased IL‐35 levels in patients with colorectal cancer, demonstrating that IL‐35 can exert antitumor effects by suppressing *β*‐catenin expression [40]. Additionally, Long et al. found that overexpression of IL‐35 in human cancer cells inhibits cell growth in vitro and induces G1 phase cell cycle arrest [41]. This observation, consistent with our study, may explain the reduced IL‐35 levels in malignant SGTs with higher growth rates. Interestingly, another study discovered that IL‐35 produced by tumor cells can promote tumor growth through angiogenesis within the tumor microenvironment [25]. However, a study by Jiang et al. reported that IL‐35 inhibits angiogenesis and inflammation in RA by reducing vascular endothelial growth factor (VEGF) expression [42]. This mechanism may also account for the reduced IL‐35 levels observed in malignant SGTs, further supporting our study’s findings.

In this study, IL‐35 levels were significantly lower in patients with malignant SGTs compared to the control group, but no difference was observed between benign and malignant tumors. Several factors may explain these findings. In healthy individuals, IL‐35 is primarily produced by peripheral Tregs. In the tumor microenvironment, cancer cells or tumor stroma may release inhibitory signals that reduce IL‐35 production. Both benign and malignant SGTs may share similar cytokine patterns, as they originate from the same salivary gland epithelium and activate common innate immune mechanisms.

One limitation of the current study was the relatively modest sample size. Additionally, the retrospective nature of the study limited access to complete patient information, as missing data in the records made it impossible to gather specific details. Consequently, key variables like tumor grade and size were omitted from the analysis for many patients due to the lack of available data.

## 5. Conclusions

In conclusion, while IL‐35 levels are significantly reduced in malignant SGTs, its modest diagnostic performance highlights the need for multimodal approaches such as combining IL‐35 with established biomarkers or imaging techniques. Further validation in larger cohorts is essential to assess its clinical utility as part of a biomarker panel. Additionally, IL‐35 levels were not influenced by tumor origin, age, or gender. In OSCC patients, the lack of significant difference from healthy controls and absence of correlation with tumor characteristics suggest that IL‐35 is unlikely to play a significant role in OSCC pathogenesis or prognosis.

**Nomenclature**
BCCs: breast cancer cells
EndMT: endothelial‐to‐mesenchymal transition
IL‐35: interleukin‐35
LPS: lipopolysaccharide
NSCLC: non‐small‐cell lung cancer
OSCC: oral squamous cell carcinoma
pSS: primary Sjögren’s syndrome
ROC: receiver operating characteristic
RA: rheumatoid arthritis
SGTs: salivary gland tumors
SS: Sjögren’s syndrome

## Ethics Statement

This study was conducted after obtaining approval from the Ethics Committee of Shiraz University of Medical Sciences (IR.SUMS.DENTAL.REC.1400.089 and IR.SUMS.DENTAL.REC.1400.088). All patients provided written informed consent after being fully informed about the study procedures. All experiments were performed in accordance with the Declaration of Helsinki.

## Consent

The authors have nothing to report.

## Disclosure

All authors gave final approval and agreed to be accountable for all aspects of the work, ensuring integrity and accuracy.

## Conflicts of Interest

The authors declare no conflicts of interest.

## Author Contributions

The authors M.Z., B.K., A.G., M.J.F., and J.G. contributed to the study’s design, acquisition, analysis, and interpretation of data; drafted the manuscript; and critically revised the manuscript. The authors S.M., N.H., and F.K. contributed to the study’s design, analysis, and interpretation of data and critically revised the manuscript.

## Funding

This work was supported by the Shiraz University of Medical Sciences, 10.13039/501100004320, 24328 and 24058.

## Data Availability

The datasets used and/or analyzed during the current study are available from the corresponding author on reasonable request.
